# The Impact of Alcohol Consumption on Cognitive Impairment in Patients With Diabetes, Hypertension, or Chronic Kidney Disease

**DOI:** 10.3389/fmed.2022.861145

**Published:** 2022-06-03

**Authors:** Fu-Shun Yen, Shiow-Ing Wang, Shih-Yi Lin, Yung-Hsiang Chao, James Cheng-Chung Wei

**Affiliations:** ^1^Dr. Yen’s Clinic, Taoyuan, Taiwan; ^2^Center for Health Data Science, Department of Medical Research, Chung Shan Medical University Hospital, Taichung City, Taiwan; ^3^Department of Medical Research, Center for Health Data Science, Chung Shan Medical University Hospital, Taichung City, Taiwan; ^4^Department of Medicine, School of Medicine, National Yang Ming Chiao Tung University, Taipei, Taiwan; ^5^Institute of Medicine, Chung Shan Medical University, Taichung City, Taiwan; ^6^Department of Allergy, Immunology and Rheumatology, Chung Shan Medical University Hospital, Taichung City, Taiwan; ^7^Graduate Institute of Integrated Medicine, China Medical University, Taichung City, Taiwan

**Keywords:** cognitive impairment, alcohol drinking, diabetes, hypertension, chronic kidney disease

## Abstract

To investigate the impact of alcohol use on the risk of cognitive impairment in older adults with chronic illness, we used the Digit Symbol Substitution Test (DSST) to evaluate cognitive function in older adults (≥ 60 years) in the National Health and Nutrition Examination Survey. Participants were categorized as light drinkers, moderate and heavy drinkers. Logistic regression analyses were used to explore associations between cognitive impairment and alcohol drinking in patients with or without diabetes, hypertension, and chronic kidney disease (CKD). Multivariate analysis showed that alcohol heavy drinkers was significantly associated with a higher risk of cognitive impairment in patients with **hypertension** (aOR 6.089, 95% CI 1.318–28.13) and CKD (aOR 6.324, 95% CI 1.158–34.52) compared with light drinkers. The dose-response analyses revealed that moderate to heavy alcohol use was associated with a higher risk of cognitive decline in patients with diabetes and CKD, heavy drinking increased the risk of cognitive impairment in patients with hypertension. The impacts of alcohol drinking on cognitive impairment are significantly different in patients with different comorbidities.

## Introduction

Cognition refers to skills, such as learning, comprehension, memory, expression, language, communication, and execution (1). Cognitive impairment is a deficit of key brain functions, such as concentration, learning, memory, and decision-making. It ranges from mild to severe, and severe impairment may affect daily life and independence (2). The rate of cognitive impairment and dementia increases with age (3); generally, about 4–19% of 65-year-old persons have cognitive impairment (depending on the definition) (2). In addition to aging, genes, lifestyle, environmental risk factors, and chronic illnesses may affect cognitive function (2, 3). Cognitive impairment and dementia are significant causes of morbidity and mortality worldwide, imposing a heavy economic burden on patients, families, and caregivers. Identification of modifiable risk factors for cognitive impairment may help attenuate the global burden of dementia.

Alcohol drinking can influence mood and cognition. Several studies have shown that chronic heavy alcohol drinking may lead to thiamine deficiency, ataxia, confusion, and oculomotor changes (Wernicke-Korsakoff syndrome) (4). Most, but not all, studies have shown that heavy alcohol drinking may increase the risk of cognitive impairment, but light to moderate amounts of alcohol consumption may attenuate the risk of cognitive impairment. Large amounts of alcohol may be metabolized to acetaldehyde, resulting in brain injury or nutritional deficiency and influencing neurocircuits. Light to moderate alcohol use may increase blood high-density lipoprotein (HDL), decrease oxidant stress, thrombosis, coagulation, and cardiovascular diseases (5).

The number of people with diabetes, hypertension, and chronic kidney disease (CKD) has increased recently, perhaps due to lifestyle, dietary changes and increased life expectancy worldwide. Approximately 22.8, 59.6, and 34.1% of 65 years and older persons have diabetes, hypertension, and CKD (6, 7). Hyperglycemia and insulin resistance increase the risk of cognitive impairment in patients with diabetes (2). The risk of new-onset cognitive impairment (up to 60%) is higher in persons with diabetes than those without diabetes (8). Hypertension, mediated by chronic mechanical stress, can significantly increase the risk of stroke and cerebral white matter lesions, leading to cognitive impairment (9). Studies have shown CKD (with accompanying risks of cardiovascular diseases and uremic metabolites) as a risk factor for cognitive dysfunction (10). Diabetes, hypertension, and CKD potentially contribute to a substantial proportion of cognitive impairment worldwide.

Many older people also have chronic diseases (11). People with more illnesses are more likely to develop cognitive impairment due to a lack of resilience and repair of cognitive function (2). These chronic diseases may compound the global burden of cognitive impairment. The relationship between alcohol drinking and cognitive change may be mediated or moderated by the presence of chronic illness. However, studies on the impact of alcohol use on cognitive function in patients with chronic diseases are scarce (12); and there is a close association among diabetes, hypertension, CKD and cognitive impairment. Therefore, we designed this study to investigate the effect of alcohol drinking on cognitive impairment in older people with diabetes, hypertension, or CKD.

## Materials and Methods

### Data Source

This study used the dataset from the National Health and Nutrition Examination Survey (NHANES). Since the early 1960s, the NHANES program has been conducted as a series of surveys focusing on different health topics (13). The sample population for the NHANES survey is the non-institutionalized United States population. Further information on background, design, and operation is available on the NHANES website.^1^

### Ethics Statement

The NHANES data was de-identified and encrypted, and all participants in NHANES provided written informed consent consistent with the approval by the National Center for Health Statistics Institutional Review Board. Review and approval by the Institutional Review Board and the informed consent signed by participants were waived in the NHANES data analysis. In addition, the present study was approved under the authority of the Institutional Review Board of Chung Shan Medical University Hospital (CSMUH No: CS1-22004).

### Study Subjects

Participants aged ≥ 60 years received the cognitive functioning assessment using Digit Symbol Substitution Test (DSST) (14) and completed the questionnaire of alcohol consumption in the NHANES cycles of 1999–2002 and 2011–2014. We excluded participants with incomplete DSST assessments or without alcohol drinking information. Patients diagnosed with brain tumors were also excluded. Supplementary Figure 1 depicts the selection process.

### Study Variables

#### Cognitive Impairment

Cognitive impairment was defined as a DSST score below the lowest quartile. The DSST requests the participant to code a series of symbols in 120 s with accuracy. This exercise demands sustained attention, response speed, visual-spatial skills, associative learning, and memory, providing a sensitive measure of cognitive function (14).

#### Alcohol Consumption

According to the Substance Abuse and Mental Health Services Administration (SAMHSA) criteria, and the limitation of NHANES questionnaire design, alcohol drinkers were classified into three groups: (1) Light drinkers (less or equal 1 drink/day); (2) Moderate drinkers (2–4 drinks/day in males, 2–3 drinks/day in females); (3) Heavy drinkers (≥ 5 drinks/day in males, ≥ 4 drinks/day in females) (15). We obtained information on alcohol consumption from the alcohol use questionnaire to estimate the average alcohol intake per day, and stratified the drinking dose based on the question:” In the past 12 months, on those days that you drank alcoholic beverages, on the average, how many drinks did you have?” One equivalent of alcoholic drink [12 fluid ounces of regular beer (5% alcohol), 5 fluid ounces of wine (12% alcohol), or 1.5 fluid ounces of 80 proof distilled spirits (40% alcohol)] contain about 14 g of alcohol.

#### Demographic

We obtained information on age, sex, marital status, race/ethnicity, education levels, and poverty income ratio (a ratio of family income to poverty threshold) from the NHANES database. Racial identity was grouped as non-Hispanic White, non-Hispanic Black, other Hispanic, Mexican American, and others (including multi-racial). Marital status was classified as married/living with a partner, widowed/divorced/separated, and never married. Educational status was collated as under 12th grade, high school graduate, and college or above. Poverty income ratio (a ratio of family income to poverty threshold) was categorized as ≤ 1.30, 1.31–3.50, > 3.50 (richest).

#### Lifestyle

Smoking status was categorized as: (1) non-smokers: who have never smoked 100 cigarettes during their lifetime, and (2) smokers: who have smoked at least 100 cigarettes during their lifetime. We obtained information on physical activity from the questionnaire section, which covers an extensive array of questions related to leisure-time activities, daily activities, and sedentary activities at home. Metabolic equivalent (MET) scores of activities were calculated for each subject. Physical activities were divided into two groups: active (MET ≥ 500 per week) and inactive [MET < 500 per week (16)]. Body mass index (BMI): BMI (weight/height^2^) was calculated during physical examinations at the NHANES mobile examination center (MEC). According to the World Health Organization (WHO) criteria, BMI was classified into four groups: underweight (< 18.5 Kg/m^2^), normal (18.5∼24.9 Kg/m^2^), overweight (25∼29.9 Kg/m^2^), and obese (≥ 30.0 Kg/m^2^). Difficulties in attending social events were self-reported data on functional limitations defined by the question “By yourself and without using any special equipment, how much difficulty do you have participating in social activities [visiting friends, attending clubs or meetings or parties]?” The responses were categorized into no difficulty and difficulty (including some difficulty, much difficulty, and unable to do).

#### Comorbidities

Comorbidities, including diabetes, hypertension, and stroke, were self-reported by patients using NHANES interviewer-administered questionnaires and defined by the question “Have you ever been told by a doctor or other health professional that you had …?” Chronic Kidney Disease (CKD) was defined by urine albumin and creatinine ratio (ACR) ≥ 30 mg/g.

### Statistical Analysis

Analysis was aimed to explore the relationship between alcohol consumption and factors associated with cognitive impairment in different comorbidities in patients with diabetes, hypertension, or CKD.

Data on basic characteristics were expressed as unweighted counts (weighted%) for categorical variables and mean ± standard error for continuous variables. The Chi-square test was used to determine the differences in categorical variables, and differences in continuous variables were examined using the Complex Samples General Linear Model (CSGLM). Univariate and multivariate logistic regression analyses were performed to investigate associations between cognitive impairment and alcohol drinking. We selected variables with a significant *p*-value < 0.05 by univariate analysis and evaluated them using a multivariate logistic regression model. Interaction analysis was performed between alcohol drinking and related factors. Furthermore, to better delineate the dose-response relationship between alcohol use and cognitive impairment, the study subjects were divided into 1, 2, 3–4, 5–6, 7–8, 9–10, and 10 + drinks. Odds ratios (OR) and 95% confidence intervals (CI) were stated with different models.

All analyses included full sample MEC exam weight (WTMEC4YR for 1999–2002, WTMEC2YR for 2011–2014), stratum, and primary sampling units (PSU) per recommendations from the National Center for Health Statistics (NCHS) to perform complex sampling design analysis for address oversampling, non-response, non-coverage, and provide nationally representative estimates. Currently, NCHS recommends the use of Taylor Series Linearization methods for variance estimation in all NHANES surveys. In the present study, statistical analyses were performed using the statistical software package SPSS complex sample module version 22.0 (IBM Corp., Armonk, NY), which can be used to estimate sampling errors by the Taylor series (linearization) method. All statistical assessments were two-sided and evaluated at the 0.05 level of significance.

## Results

### Study Population Characteristics

A total of 3,006 participants were eligible for the present study. Using the NHANES sample weight, the analytic sample size in the present study represented 27,162,844 non-institutionalized participants from the United States. Basic characteristics of the study subjects stratified by diabetes, hypertension, or CKD are shown in Supplementary Tables 1–3. A higher percentage of cognitive impairment, more light drinker, lower mean DSST scores (48.71 ± 0.777 vs. 54.73 ± 0.565, *p* < 0.001) was present in patients with diabetes than those without diabetes (Supplementary Table 1). Similar patterns also appeared in hypertension and CKD groups (Supplementary Tables 2, 3).

### Factors Associated With Cognitive Impairment

In the diabetes group (Supplementary Table 4), after adjusting significant factors in univariate regression, the results of multivariate logistic regression indicated that moderate or heavy drinker vs. light drinker were positively associated with the risk of cognitive impairment, but not reach the significance [adjusted odds ratio (aOR) = 1.233 and 3.237, respectively]. Moreover, the following factors were significantly positively associated with cognitive impairment: age (70 + y vs. 60–69 y), race (other Hispanic, Mexican American, and non-Hispanic Black vs. non-Hispanic White), education (under 12th grade vs. college or above), attend social events difficulty and CKD.

In the hypertension group, the results of multivariate logistic regression indicated that heavy vs. light drinkers were significantly positively associated with the risk of cognitive impairment (aOR = 6.089, 95% CI = 1.318–28.13). Age, male, race, education (under 12th grade, high school graduate vs. college or above), marital status, income ratio (≤ 1.30 vs. ≥ 3.5), physical activity (inactive vs. active), social attendance (difficulty vs. no difficulty), and CKD were significantly positively associated with cognitive impairment (Supplementary Table 5). The interaction term for alcohol consumption and physical activity in the multivariate model was also significant. In patients without hypertension, moderate or heavy alcohol consumption vs. light drinker were negatively associated with the risk of cognitive impairment, but not reach the significance (aOR = 0.491, and 0.383, respectively).

In the CKD group, heavy vs. light drinkers were significantly positively associated with the risk of cognitive impairment (aOR = 6.324, 95% CI = 1.158–34.52). Age, race (Black vs. White), education, and difficulty attending social events showed a significant positive association with cognitive impairment (Supplementary Table 6).

### Dose-Response Relationship Between Alcohol Consumption and Cognitive Impairment

Supplementary Table 7 describes the characteristics of study subjects stratified by alcoholic drinks in diabetes, hypertension, and CKD groups. The diabetes group showed two peaks of DSST scores. DSST scores increased with alcohol dose up to 1 drink, and then gradually decreased. The second peak occurred in 9–10 drinks. The hypertension group showed a similar pattern between alcohol intake and cognitive impairment. DSST scores increased with increased alcohol dose, up to 2 drinks and then gradually decreased. The score of DSST of CKD group indicate sliding all the way down, but the number of people with alcohol intake ≥ 7 drinks was small, and the value was unstable.

**FIGURE 1:**
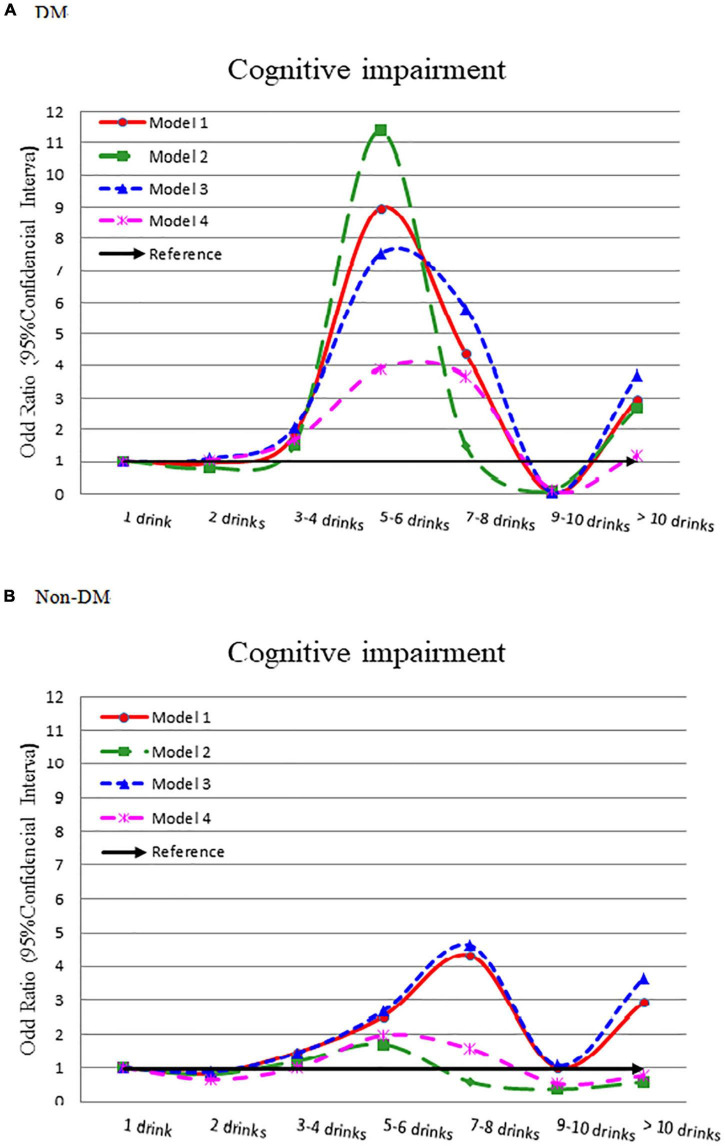
Odds ratio for cognitive impairment by different alcohol consumption categories-stratified by diabetes. **(A)** With diabetes and **(B)** without diabetes. Model 1: crude OR (95%CI). Model 2: adjusted for demographic variables. Model 3: adjusted for comorbidity variables. Model 4: adjusted for the significant variables in Supplementary Table 4.

Table 1 compiles the results of multivariate logistic regression from 4 models depicted in the diabetes group. Compared to light drinker (1 drink), 2 drinks had a lower risk of cognitive impairment (crude OR = 0.920), but 3–4, 5–6, 7–8 drinks had a higher risk of cognitive impairment (OR = 1.844, 8.966, and 4.377, respectively). We observed a bell-shaped curve between alcohol intake and cognitive impairment (Figure 1A) that remained consistent even after adjusting for demographic variables (Model 2), comorbidity variables (Model 3), and all significant variables (Table 1) (Model 4). In patients without diabetes, we noted a smoother wave between alcohol intake and cognitive impairment (Figure 1B). Compared to light drinker (1 drink), 2 drinks had lower odds of cognitive impairment (adjusted OR = 0.648, 95% CI = 0.432–0.970).

**TABLE 1:** Odd ratio for cognitive impairment by different alcohol consumption categories-stratified by diabetes.

Alcohol consumption	With diabetes				Without diabetes			
	Model 1	Model 2	Model 3	Model 4^a^	Model 1	Model 2	Model 3	Model 4^b^
		
	OR (95%CI)	aOR (95%CI)	aOR (95%CI)	aOR (95%CI)	OR (95%CI)	aOR (95%CI)	aOR (95%CI)	aOR (95%CI)
1 drink	Reference	Reference	Reference	Reference	Reference	Reference	Reference	Reference
2 drinks	0.920 (0.517–1.639)	0.766 (0.382–1.537)	1.093 (0.609–1.961)	1.045 (0.561–1.947)	0.837 (0.613–1.144)	0.802 (0.566–1.136)	0.875 (0.629–1.219)	0.648 (0.432–0.970)
3–4 drinks	1.844 (0.936–3.632)	1.515 (0.697–3.292)	2.069 (1.057–4.050)	1.684 (0.815–3.479)	1.467 (1.041–2.066)	1.196 (0.715–1.998)	1.445 (1.001–2.087)	1.000 (0.562–1.779)
5–6 drinks	8.966 (2.749–29.23)	11.36 (1.770–72.95)	7.500 (2.466–22.81)	3.909 (0.461–33.12)	2.527 (1.317–4.851)	1.711 (0.672–4.354)	2.674 (1.368–5.228)	1.981 (0.521–7.543)
7–8 drinks	4.377 (0.690–27.75)	1.490 (0.243–9.123)	5.810 (1.079–31.29)	3.656 (0.779–17.16)	4.337 (1.313–14.31)	0.583 (0.157–2.157)	4.638 (1.419–15.15)	1.580 (0.318–7.852)
9–10 drinks	0.043 (0.003–0.550)	0.102 (0.008–1.310)	0.055 (0.004–0.732)	0.134 (0.011–1.698)	1.008 (0.133–7.665)	0.362 (0.060–2.179)	1.068 (0.124–9.218)	0.536 (0.055–5.260)
10 + drinks	2.966 (0.560–15.71)	2.678 (0.197–36.48)	3.690 (0.749–18.18)	1.178 (0.294–4.715)	2.923 (0.602–14.19)	0.579 (0.068–4.921)	3.645 (0.658–20.19)	0.763 (0.077–7.609)

*Model 1: crude OR (95%CI). Model 2: adjusted for demographic variables. Model 3: adjusted for comorbidity variables. Model 4^a^: adjusted for all significant variables in Supplementary Table 4 (age, race, education, social attendance, CKD). Model 4^b^: adjusted for all significant variables in Supplementary Table 4 (age, gender, race, education, income, physical activities, social attendance, hypertension, CKD).*

**FIGURE 2:**
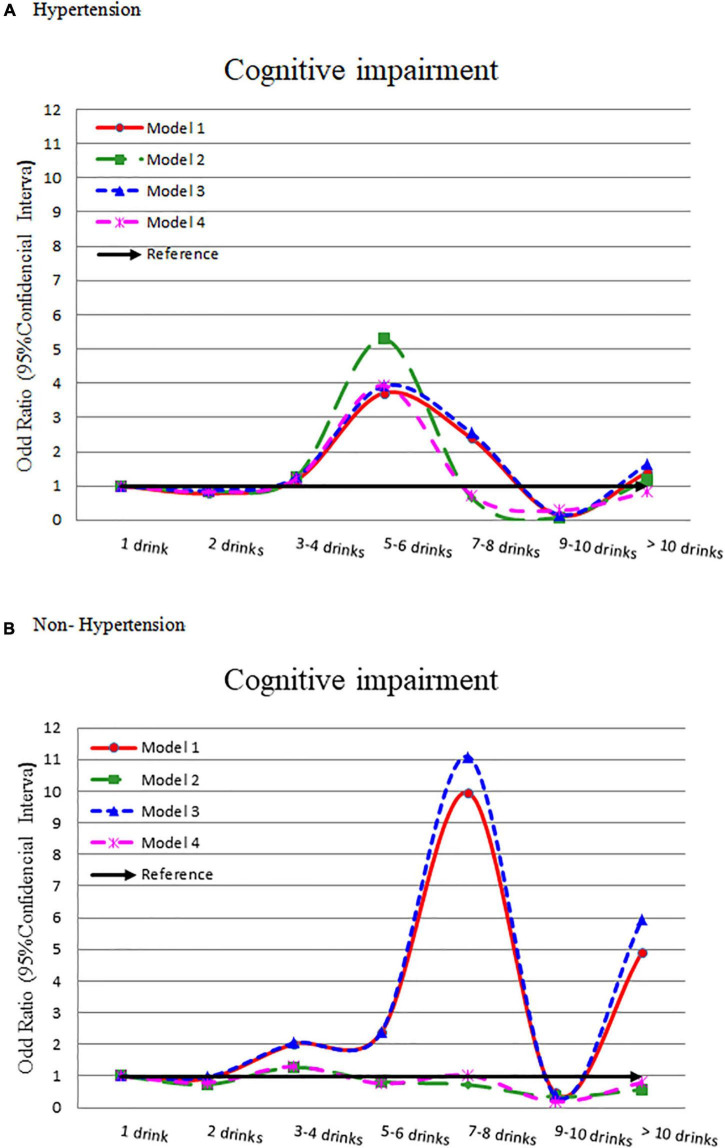
Odds ratio for cognitive impairment by different alcohol consumption categories-stratified by hypertension. **(A)** With hypertension and **(B)** without hypertension. Model 1: crude OR (95%CI). Model 2: adjusted for demographic variables. Model 3: adjusted for comorbidity variables. Model 4: adjusted for the significant variables in Supplementary Table 5.

The hypertension group showed a lower flat bell shape association between alcohol intake and cognitive impairment (Figure 2A). Compared to light drinker (1 drink), 5–6 drinks had a significantly higher odds of cognitive impairment (OR = 3.714, 95% CI = 1.610–8.565). This association was consistent after adjusting for other confounders (OR = 5.288, 3.921, and 3.934, respectively) (Table 2). The odds ratio for cognitive impairment by different alcohol consumption categories was flat in those without hypertension (Figure 2B).

**TABLE 2:** Odd ratio for cognitive impairment by different alcohol consumption categories–stratified by hypertension.

Alcohol consumption	With hypertension				Without hypertension			
	Model 1	Model 2	Model 3	Model 4^a^	Model 1	Model 2	Model 3	Model 4^b^
		
	OR (95%CI)	aOR (95%CI)	aOR (95%CI)	aOR (95%CI)	OR (95%CI)	aOR (95%CI)	aOR (95%CI)	aOR (95%CI)
1 Drink	Reference	Reference	Reference	Reference	Reference	Reference	Reference	Reference
2 Drinks	0.782 (0.541–1.131)	0.846 (0.544–1.314)	0.871 (0.588–1.290)	0.823 (0.525–1.291)	0.926 (0.596–1.438)	0.736 (0.459–1.182)	0.974 (0.607–1.562)	0.791 (0.460–1.359)
3–4 Drinks	1.172 (0.798–1.722)	1.267 (0.707–2.269)	1.251 (0.839–1.864)	1.203 (0.630–2.295)	2.017 (1.235–3.295)	1.275 (0.555–2.929)	2.045 (1.210–3.455)	1.303 (0.581–2.921)
5–6 Drinks	3.714 (1.610–8.565)	5.288 (1.705–16.39)	3.921 (1.822–8.438)	3.934 (1.065–14.53)	2.376 (1.198–4.714)	0.828 (0.402–1.705)	2.382 (1.222–4.643)	0.766 (0.371–1.579)
7–8 Drinks	2.385 (0.554–10.27)	0.658 (0.110–3.927)	2.538 (0.652–9.875)	0.731 (0.122–4.398)	9.955 (1.883–52.61)	0.725 (0.120–4.392)	11.09 (2.332–52.80)	1.026 (0.179–5.879)
9–10 Drinks	0.141 (0.056–0.352)	0.082 (0.005–1.248)	0.161 (0.052–0.495)	0.284 (0.018–4.442)	0.468 (0.044–4.987)	0.339 (0.056–2.055)	0.387 (0.031–4.796)	0.189 (0.016–2.207)
10 + drinks	1.398 (0.223–8.782)	1.181 (0.193–7.219)	1.622 (0.311–8.452)	0.820 (0.107–6.306)	4.906 (1.353–17.79)	0.571 (0.040–8.065)	5.937 (1.567–22.48)	0.796 (0.057–11.14)

*Model 1: crude OR (95%CI). Model 2: adjusted for demographic variables. Model 3: adjusted for comorbidity variables. Model 4^a^: adjusted for all significant variables in Supplementary Table 5 (age, gender, race, education, marriage, income, social attendance, CKD). Model 4^b^: adjusted for all significant variables in Supplementary Table 5 (age, gender, race, education, income, social attendance, CKD).*

**FIGURE 3:**
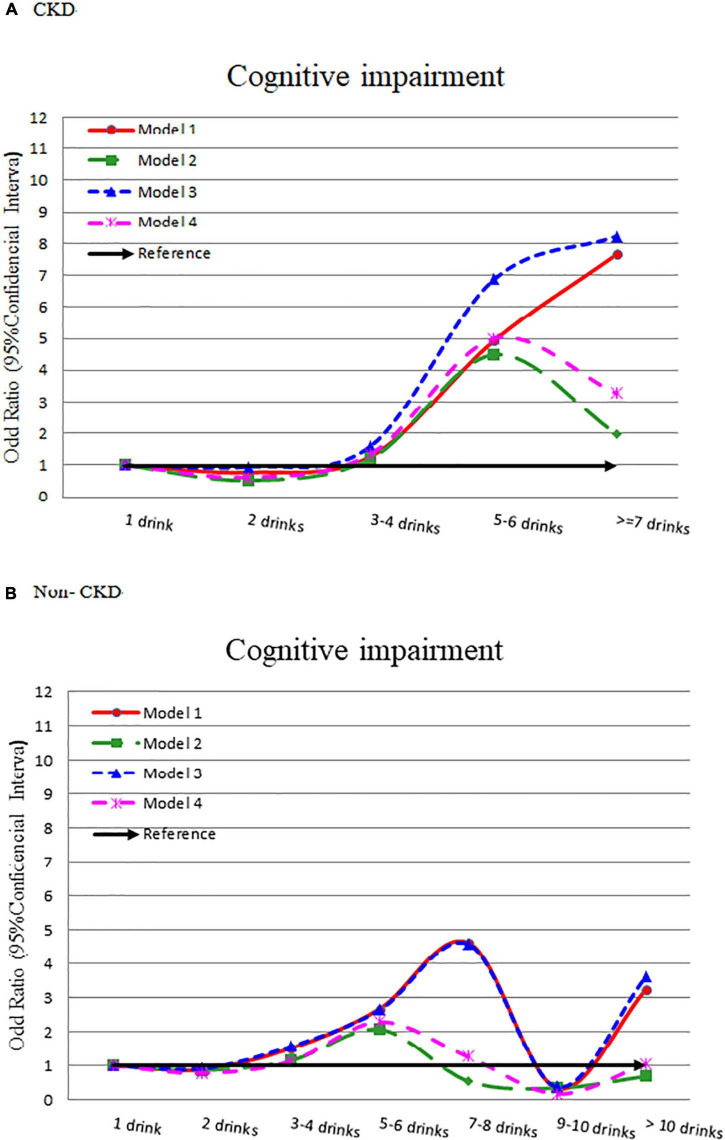
Odds ratio for cognitive impairment by different alcohol consumption categories-stratified by CKD. **(A)** With CKD and **(B)** without CKD. Model 1: crude OR (95%CI). Model 2: adjusted for demographic variables. Model 3: adjusted for comorbidity variables. Model 4: adjusted for all significant variables in Supplementary Table 6.

In the CKD group, it showed a slight J-shaped association between alcohol intake and cognitive impairment (Figure 3). The risk of cognitive impairment increased with alcohol consumption (adjusted OR = 0.618, 1.354, 4.988, and 3.266, respectively) (Supplementary Table 8). In the without CKD group, after adjusting for other influencing factors, there was no significant difference in the risk of cognitive impairment by different alcohol consumption amount.

## Discussion

This study demonstrated the effect of alcohol drinking on cognitive impairment is different in older people comorbid with diabetes, hypertension or CKD. In patients with diabetes and CKD, moderate to heavy amounts of alcohol drinking was associated with a higher risk of cognitive decline; in patients with hypertension, heavy drinking was associated with increased risk of cognitive impairment. While in patients without diabetes, hypertension or CKD, alcohol drinking seemed to have a neutral effect on cognitive function.

Diabetes is a long-known risk factor for cognitive impairment and dementia (2, 8). Chronic hyperglycemia can produce advanced glycation end-products, increase oxidative stress and chronic low-grade inflammation, cause brain microangiopathy and injury (17). Reports also suggest that insulin resistance reduces regional cerebral glucose metabolism and utilization (18). Our study showed that in persons with diabetes, moderate to heavy alcohol drinking was associated with a higher risk of cognitive impairment compared with light drinkers. A Zutphen study disclosed that alcohol consumption was associated with a lower risk of cognitive decline, but older adults who drank ≧3 drinks were associated with higher risk of cognitive impairment than those who drank 1–2 drinks per day in patients with diabetes or cardiovascular diseases (12). Moderate to heavy alcohol drinking may aggravate brain injury caused by diabetes. However, further research is needed to confirm this finding. Therefore, old people with diabetes may be warned not to drink much alcohol.

Hypertension is the main attributor to global dementia (2). Many studies have shown that midlife high blood pressure is a risk factor for late-life cognitive impairment and dementia. Longstanding hypertension may result in atherosclerosis, arterial stiffness, and hypoperfusion of the brain, which can compromise cerebral oxygen supply, leading to neuronal injury (19). Our study showed that moderate to heavy alcohol drinking was associated with a higher risk of cognitive impairment than light drinking. Studies show that heavy alcohol drinking elevates acetaldehyde, which is neurotoxic (20). Alcohol can modify calcium channels in the brain and impair several neurocircuits. Ethanol can block N-methyl-D-aspartate (NMDA) receptors and increase glutamate release with neurotoxic effects (4, 21). Heavy alcohol drinking increased blood pressure and the incidence of hypertension (22, 23), which could have resulted in brain damage in our patients with hypertension. Our results suggested that the protective magnitude of light alcohol drinking on cognitive function could not prevent brain damage caused by hypertension and increased the risk of cognitive impairment. Older adults with hypertension should have small amounts of alcohol with exercise, intellectual stimulation, and leisure time to attenuate the risk of cognitive decline (2).

Recently, several studies have found that chronic kidney disease may increase the risk of cognitive impairment (10, 24). Patients with CKD have disproportionate levels of cerebrovascular disease, high prevalence of diabetes, hypertension, dyslipidemia, and uremic metabolites that can lead to cognitive impairment or dementia. Our study revealed that moderate to heavy alcohol drinking in patients with CKD was associated with increased risk of cognitive impairment. Old people with CKD should only drink small amounts of alcohol and moderate themselves to healthier living in dietary, physical, and mental perspectives.

This study had some limitations. First, diabetes and hypertension were reported by participants instead of objective measures. Misclassification bias could exist. Participants with chronic kidney disease usually have no symptoms and medication, they may not know their renal disease. The NHANES survey gets the information of chronic kidney disease through laboratory tests and not by reports of participants. We used different methods to obtain data of the three chronic diseases, which may influence outcomes of this research. Second, alcohol consumption status was asked twice in each cycle of NHANES, the recall bias may exist, but evidence has revealed that self-report of alcohol use may be a reliable and valid approach to measure alcohol consumption (25). Third, because the NHANES is a cross-sectional dataset, we could not investigate the impact of different durations and patterns of alcohol use in these patients. Fourth, we adopted the DSST to assess cognitive dysfunction. Although DSST is a sensitive method for cognitive decline (1), it does not provide all the different faces of cognitive function. However, a change in one cognitive domain may presage a change in other cognition domains. Fifth, NHANES participants resided in communities, not institutions; those who could not take the DSST test were excluded. Therefore, this study may have included relatively healthy people and is not representative of the entire population. Finally, this cross-sectional study had unknown and unobserved biases. Prospective studies abiding by stringent and rigorous methodologies are warranted to verify our results.

Our study demonstrated that the impacts of alcohol drinking on cognitive impairment are significantly different in patients with various comorbidities. Since older people are often accompanied by one or more chronic diseases, the results of this study can provide an empirical evidence for clinicians or health professionals to make alcohol drinking recommendations. Older adults with chronic illnesses should have adequate amounts of alcohol drinking to mitigate the future risks of cognitive decline.

## Data Availability Statement

The original contributions presented in this study are included in the article/Supplementary Material, further inquiries can be directed to the corresponding author/s.

## Ethics Statement

Ethical review and approval was not required for the study on human participants in accordance with the local legislation and institutional requirements. Written informed consent for participation was not required for this study in accordance with the national legislation and the institutional requirements.

## Author Contributions

F-SY and JC-CW: conceptualization. S-IW and S-YL: methodology. S-IW and Y-HC: software. S-IW and JC-CW: validation and project administration. S-IW: formal analysis and visualization. F-SY and S-YL: investigation. S-IW and F-SY: data curation. F-SY, S-YL, and S-IW: writing—original draft preparation. JC-CW and Y-HC: writing—review and editing. JC-CW: supervision and resources. All authors have read and agreed to the published version of the manuscript.

## Conflict of Interest

The authors declare that the research was conducted in the absence of any commercial or financial relationships that could be construed as a potential conflict of interest.

## Publisher’s Note

All claims expressed in this article are solely those of the authors and do not necessarily represent those of their affiliated organizations, or those of the publisher, the editors and the reviewers. Any product that may be evaluated in this article, or claim that may be made by its manufacturer, is not guaranteed or endorsed by the publisher.

## Footnotes

1
https://www.cdc.gov/nchs/nhanes/about_nhanes.htm

## Abbreviations

DSST: digit symbol substitution test
MET: metabolic equivalent
CKD: chronic kidney disease
DM: diabetes mellitus
NMDA: N-methyl -D-aspartate.
