# Corrigendum: The impact of alcohol consumption on cognitive impairment in patients with diabetes, hypertension, or chronic kidney disease

**DOI:** 10.3389/fmed.2022.1027146

**Published:** 2022-09-23

**Authors:** Fu-Shun Yen, Shiow-Ing Wang, Shih-Yi Lin, Yung-Hsiang Chao, James Cheng-Chung Wei

**Affiliations:** ^1^Dr. Yen's Clinic, Taoyuan, Taiwan; ^2^Department of Medical Research, Center for Health Data Science, Chung Shan Medical University Hospital, Taichung City, Taiwan; ^3^Center for Geriatrics and Gerontology, Taichung Veterans General Hospital, Taichung, Taiwan; ^4^Department of Medicine, School of Medicine, National Yang Ming Chiao Tung University, Taipei, Taiwan; ^5^Institute of Medicine, Chung Shan Medical University, Taichung City, Taiwan; ^6^Department of Allergy, Immunology and Rheumatology, Chung Shan Medical University Hospital, Taichung City, Taiwan; ^7^Graduate Institute of Integrated Medicine, China Medical University, Taichung City, Taiwan

**Keywords:** cognitive impairment, alcohol drinking, diabetes, hypertension, chronic kidney disease

In the original article, there is an error in the author list, and authors “Fu-Shun Yen^1^, Shiow-Ing Wang^2^” were erroneously listed. The correction appears below.

“Fu-Shun Yen^1^^†^, Shiow-Ing Wang^2^^†^

^†^These authors have contributed equally to this work”

The authors apologize for this error and state that this does not change the scientific conclusions of the article in any way. The original article has been updated.

## Publisher's note

All claims expressed in this article are solely those of the authors and do not necessarily represent those of their affiliated organizations, or those of the publisher, the editors and the reviewers. Any product that may be evaluated in this article, or claim that may be made by its manufacturer, is not guaranteed or endorsed by the publisher.

