# Supplementary material for: The Impact of Alcohol Consumption on Cognitive Impairment in Patients With Diabetes, Hypertension, or Chronic Kidney Disease
Source: Front Med (Lausanne). 2022 Jun 3;9:861145. doi: 10.3389/fmed.2022.861145 (PMC9203733; doi:10.3389/fmed.2022.861145)
Supplement: Supplementary file 1 [file Table_1.DOCX]

Supplementary Material

# Supplementary Tables

**Table S1.** Characteristics of participants (unweighted sample sizes and weighted %) according to cognitive impairment-diabetes.

| **Variables** | | **With Diabetes**  **(n=573/N=4,597,888)** | **Without Diabetes**  **(n=2,432/N=22,548,227)** | **P value** |
| --- | --- | --- | --- | --- |
| Cognitive function | |  |  |  |
|  | Impairment | 196 (22.0) | 651 (15.5) | 0.003 |
|  | DSST ^1^ score | 48.71 ± 0.777 | 54.73 ± 0.565 | <0.001 |
| Alcohol consumption | |  |  |  |
|  | Alcohol (drinks/day), mean±SE^2^ | 1.72 ± 0.088 | 1.76 ± 0.034 | 0.746 |
|  | Light drinkers | 335 (64.7) | 1318 (56.4) | 0.004 |
|  | Moderate drinkers | 193 (30.0) | 955 (39.5) |  |
|  | Heavy drinkers | 45 (05.4) | 159 (04.0) |  |
| Demographic | |  |  |  |
|  | Age, years |  |  | 0.776 |
|  | 70+ | 239 (41.5) | 1129 (42.4) |  |
|  | 60-69 | 334 (58.5) | 1303 (57.6) |  |
|  | Gender |  |  | 0.002 |
|  | Male | 357 (58.6) | 1302 (48.0) |  |
|  | Race |  |  | <0.001 |
|  | Others (including multi-racial) | 43 (05.1) | 125 (02.7) |  |
|  | Other Hispanic | 43 (03.5) | 163 (03.3) |  |
|  | Mexican American | 90 (04.2) | 292 (02.4) |  |
|  | Non-Hispanic Black | 133 (09.2) | 357 (04.9) |  |
|  | Non-Hispanic White | 264 (78.0) | 1495 (86.8) |  |
|  | Education |  |  | 0.037 |
|  | Under 12th grade | 163 (18.6) | 582 (14.3) |  |
|  | High school graduate | 131 (25.0) | 545 (21.7) |  |
|  | College or above | 278 (56.4) | 1305 (64.0) |  |
|  | Marital status |  |  | 0.399 |
|  | Married/ Living with partner | 350 (65.5) | 1522 (69.4) |  |
|  | Widowed/ Divorced/ Separated | 189 (30.3) | 744 (27.3) |  |
|  | Never married | 28 (04.2) | 104 (03.3) |  |
|  | Income ratio |  |  | <0.001 |
|  | ≤1.30 | 136 (17.1) | 445 (12.0) |  |
|  | 1.31-3.50 | 218 (43.2) | 831 (35.4) |  |
|  | > 3.50 | 164 (39.7) | 913 (52.6) |  |
| Lifestyle | |  |  |  |
|  | Smoking |  |  | 0.014 |
|  | Smoker | 350 (61.9) | 1402 (55.5) |  |
|  | Non-smoker | 223 (38.1) | 1025 (44.5) |  |
|  | Physical activity |  |  | <0.001 |
|  | Active | 190 (39.1) | 1009 (53.1) |  |
|  | Inactive | 296 (60.9) | 903 (46.9) |  |
|  | BMI ^3^ |  |  | <0.001 |
|  | Underweight | 2 (00.2) | 37 (01.7) |  |
|  | Overweight | 206 (33.2) | 927 (38.4) |  |
|  | Obese | 268 (53.2) | 706 (29.7) |  |
|  | Normal | 86 (13.4) | 709 (30.2) |  |
|  | Attend social events |  |  | 0.001 |
|  | Difficulty | 78 (11.7) | 205 (06.6) |  |
|  | No difficulty | 481 (88.3) | 2199 (93.4) |  |
| Comorbidities | |  |  |  |
|  | Hypertension | 408 (71.8) | 1228 (49.1) | <0.001 |
|  | Stroke | 41 (07.9) | 106 (03.8) | 0.005 |
|  | CKD^4^ | 173 (24.7) | 346 (11.2) | <0.001 |

^1^ DSST: Digit Symbol Substitution Test.

^2^ SE: Standard Error.

^3^ BMI: Body Mass Index.

^4^ CKD: Chronic Kidney Disease.

**Table S2.** Characteristics of participants (unweighted sample sizes and weighted %) according to cognitive impairment-hypertension.

| **Variables** | | **With Hypertension**  **(n=1,637/N=14,380,270)** | **Without hypertension**  **(n=1,364/N=12,744,220)** | **P value** |
| --- | --- | --- | --- | --- |
| Cognitive function | |  |  |  |
|  | Impairment | 492 (19.7) | 353 (13.3) | <0.001 |
|  | DSST ^1^ score | 52.36 ± 0.665 | 55.21 ± 0.667 | 0.003 |
| Alcohol consumption | |  |  |  |
|  | Alcohol (drinks/day), mean±SE^2^ | 1.74 ± 0.053 | 1.76 ± 0.044 | 0.796 |
|  | Light drinkers | 914 (58.1) | 738 (57.7) | 0.961 |
|  | Moderate drinkers | 624 (37.8) | 523 (37.9) |  |
|  | Heavy drinkers | 99 (04.1) | 103 (04.4) |  |
| Demographic | |  |  |  |
|  | Age, years |  |  | 0.003 |
|  | 70+ | 776 (45.8) | 589 (38.1) |  |
|  | 60-69 | 861 (54.2) | 775 (61.9) |  |
|  | Gender |  |  | 0.015 |
|  | Male | 846 (47.2) | 808 (52.4) |  |
|  | Race |  |  | <0.001 |
|  | Others (including multi-racial) | 84 (02.7) | 84 (03.4) |  |
|  | Other Hispanic | 115 (03.5) | 90 (03.1) |  |
|  | Mexican American | 183 (02.5) | 197 (02.8) |  |
|  | Non-Hispanic Black | 339 (07.5) | 151 (03.5) |  |
|  | Non-Hispanic White | 916 (83.8) | 842 (87.2) |  |
|  | Education |  |  | 0.994 |
|  | Under 12th grade | 391 (15.0) | 352 (15.0) |  |
|  | High school graduate | 384 (22.4) | 293 (22.3) |  |
|  | College or above | 861 (62.6) | 719 (62.7) |  |
|  | Marital status |  |  | 0.005 |
|  | Married/ Living with partner | 982 (65.8) | 887 (72.0) |  |
|  | Widowed/ Divorced/ Separated | 549 (30.7) | 383 (24.5) |  |
|  | Never married | 71 (03.5) | 61 (03.5) |  |
|  | Income ratio |  |  | 0.015 |
|  | ≤1.30 | 339 (14.5) | 240 (10.9) |  |
|  | 1.31-3.50 | 590 (38.5) | 458 (34.7) |  |
|  | > 3.50 | 553 (46.9) | 523 (54.4) |  |
| Lifestyle | |  |  |  |
|  | Smoking |  |  | 0.271 |
|  | Smoker | 950 (57.9) | 802 (55.3) |  |
|  | Non-smoker | 686 (42.1) | 558 (44.7) |  |
|  | Physical activity |  |  | 0.003 |
|  | Active | 606 (46.7) | 591 (55.1) |  |
|  | Inactive | 730 (53.3) | 468 (44.9) |  |
|  | BMI ^3^ |  |  | <0.001 |
|  | Underweight | 15 (01.0) | 24 (02.0) |  |
|  | Overweight | 615 (37.1) | 516 (37.9) |  |
|  | Obese | 635 (41.2) | 338 (25.0) |  |
|  | Normal | 340 (20.7) | 454 (35.1) |  |
|  | Attend social events |  |  | 0.018 |
|  | Difficulty | 183 (08.7) | 99 (06.1) |  |
|  | No difficulty | 1425 (91.3) | 1252 (93.9) |  |
| Comorbidities | |  |  |  |
|  | Diabetes Mellitus | 408 (23.0) | 164 (10.2) | <0.001 |
|  | Stroke | 106 (06.2) | 41 (02.5) | 0.001 |
|  | CKD^4^ | 340 (17.1) | 179 (09.6) | <0.001 |

^1^ DSST: Digit Symbol Substitution Test.

^2^ SE: Standard Error.

^3^ BMI: Body Mass Index.

^4^ CKD: Chronic Kidney Disease.

**Table S3.** Characteristics of participants (unweighted sample sizes and weighted %) according to cognitive impairment-CKD.

| **Variables** | | **With CKD^4^**  **(n=520/N=3,635,214)** | **Without CKD^4^**  **(n=2452/N=23,222,715)** | **P value** |
| --- | --- | --- | --- | --- |
| Cognitive function | |  |  |  |
|  | Impairment | 226 (31.3) | 605 (14.0) | <0.001 |
|  | DSST ^1^ score | 46.49 ± 1.189 | 55.01 ± 0.521 | <0.001 |
| Alcohol consumption | |  |  |  |
|  | Alcohol (drinks/day), mean±SE^2^ | 1.77 ± 0.062 | 1.75 ± 0.034 | 0.713 |
|  | Light drinkers | 279 (58.3) | 1352 (57.6) | 0.453 |
|  | Moderate drinkers | 200 (36.2) | 939 (38.3) |  |
|  | Heavy drinkers | 41 (05.4) | 161 (04.1) |  |
| Demographic | |  |  |  |
|  | Age, years |  |  | <0.001 |
|  | 70+ | 290 (56.9) | 1052 (39.5) |  |
|  | 60-69 | 230 (43.1) | 1400 (60.5) |  |
|  | Gender |  |  | 0.006 |
|  | Male | 329 (58.2) | 1315 (48.6) |  |
|  | Race |  |  | 0.002 |
|  | Others (including multi-racial) | 29 (03.1) | 138 (03.1) |  |
|  | Other Hispanic | 35 (03.6) | 168 (03.2) |  |
|  | Mexican American | 70 (03.3) | 312 (02.6) |  |
|  | Non-Hispanic Black | 108 (08.7) | 378 (05.1) |  |
|  | Non-Hispanic White | 278 (81.3) | 1456 (86.0) |  |
|  | Education |  |  | 0.025 |
|  | Under 12th grade | 156 (19.9) | 587 (14.4) |  |
|  | High school graduate | 107 (22.0) | 559 (22.2) |  |
|  | College or above | 256 (58.1) | 1306 (63.4) |  |
|  | Marital status |  |  | 0.069 |
|  | Married/ Living with partner | 292 (63.3) | 1571 (70.0) |  |
|  | Widowed/ Divorced/ Separated | 190 (32.7) | 721 (26.6) |  |
|  | Never married | 26 (04.1) | 105 (03.4) |  |
|  | Income ratio |  |  | 0.001 |
|  | ≤1.30 | 133 (17.9) | 437 (11.7) |  |
|  | 1.31-3.50 | 190 (40.4) | 846 (36.0) |  |
|  | > 3.50 | 148 (41.8) | 925 (52.3) |  |
| Lifestyle | |  |  |  |
|  | Smoking |  |  | 0.003 |
|  | Smoker | 334 (65.6) | 1398 (55.0) |  |
|  | Non-smoker | 186 (34.4) | 1049 (45.0) |  |
|  | Physical activity |  |  | 0.084 |
|  | Active | 182 (45.6) | 1009 (51.5) |  |
|  | Inactive | 211 (54.4) | 972 (48.5) |  |
|  | BMI ^3^ |  |  | 0.001 |
|  | Underweight | 9 (02.2) | 30 (01.4) |  |
|  | Overweight | 167 (28.7) | 957 (38.8) |  |
|  | Obese | 196 (42.6) | 769 (32.2) |  |
|  | Normal | 129 (26.5) | 658 (27.6) |  |
|  | Attend social events |  |  | 0.001 |
|  | Difficulty | 71 (12.3) | 201 (06.3) |  |
|  | No difficulty | 440 (87.7) | 2219 (93.7) |  |
| Comorbidities | |  |  |  |
|  | Diabetes Mellitus | 173 (30.6) | 391 (14.5) | <0.001 |
|  | Hypertension | 340 (66.7) | 1277 (50.7) | <0.001 |
|  | Stroke | 47 (08.5) | 96 (03.7) | 0.001 |

^1^ DSST: Digit Symbol Substitution Test.

^2^ SE: Standard Error.

^3^ BMI: Body Mass Index.

^4^ CKD: Chronic Kidney Disease.

**Table S4.** Multivariate logistic regression analyses of risk variables for association with cognitive impairment-stratified by diabetes.

| **Variables** | | **Cognitive Impairment** | |
| --- | --- | --- | --- |
|  |  | **With Diabetes** | **Without Diabetes** |
|  |  | **aOR^3^ (95% CI^4^)** | **aOR (95% CI)** |
| Alcohol consumption (Ref ^1^= Light drinkers) | |  |  |
|  | Moderate drinkers | 1.223 (0.568-2.634) | 0.762 (0.528-1.099) |
|  | Heavy drinkers | 3.237 (0.781-13.42) | 1.490 (0.486-4.569) |
| Demographic | |  |  |
|  | Age (Ref=60-69y) |  |  |
|  | 70+ | 5.673 (2.255-14.26) | 3.136 (2.196-4.480) |
|  | Gender (Ref=Female) |  |  |
|  | Male |  | 2.891 (1.819-4.596) |
|  | Race (Ref= Non-Hispanic white) |  |  |
|  | Others (including multi-racial) | 1.730 (0.464-6.447) | 1.254 (0.685-2.297) |
|  | Other Hispanic | 11.11 (4.248-29.06) | 2.961 (1.381-6.348) |
|  | Mexican American | 4.497 (1.682-12.02) | 2.990 (1.629-5.487) |
|  | Non-Hispanic black | 5.458 (2.872-10.37) | 4.895 (3.195-7.499) |
|  | Education (Ref= College or above) |  |  |
|  | Under 12th grade | 7.376 (2.370-22.95) | 5.656 (3.501-9.137) |
|  | High school graduate | 2.669 (0.738-9.649) | 1.546 (0.979-2.440) |
|  | Marital status (Ref= Married/ Living with partner) |  |  |
|  | Widowed/ Divorced/ Separated | 1.110 (0.435-2.834) | 1.338 (0.871-2.055) |
|  | Never married | 0.452 (0.117-1.748) | 0.528 (0.256-1.087) |
|  | Income ratio (Ref= >3.50) |  |  |
|  | ≤1.30 | 0.554 (0.136-2.262) | 3.373 (1.782-6.382) |
|  | 1.31-3.50 | 0.779 (0.276-2.201) | 1.757 (1.022-3.021) |
| Lifestyle | |  |  |
|  | Smoking (Ref=Non-smokers) |  |  |
|  | Smokers | 1.862 (0.966-3.591) | 0.886 (0.665-1.182) |
|  | Physical activity (Ref= Active) |  |  |
|  | Inactive | 1.740 (0.742-4.079) | 1.511 (1.121-2.037) |
|  | Attend social events (Ref= No difficulty) |  |  |
|  | Difficulty | 2.707 (1.084-6.759) | 2.633 (1.389-4.990) |
| Comorbidities (Ref=Without) | |  |  |
|  | Hypertension |  | 1.705 (1.206-2.412) |
|  | Stroke | 1.433 (0.523-3.926) | 1.569 (0.839-2.932) |
|  | CKD ^2^ | 3.074 (1.353-6.985) | 2.531 (1.611-3.976) |

^1^ Ref: reference. ^2^ CKD: Chronic Kidney Disease. ^3^ aOR: adjusted odds ratio. ^4^ CI: Confidence interval.

**Table S5.** Multivariate logistic regression analyses of risk variables for association with cognitive impairment-stratified by hypertension.

| **Variables** | | **Cognitive Impairment** | |
| --- | --- | --- | --- |
|  |  | **With Hypertension** | **Without Hypertension** |
|  |  | **aOR^3^ (95% CI^4^)** | **aOR (95% CI)** |
| Alcohol consumption (Ref ^1^= Light drinkers) | |  |  |
|  | Moderate drinkers | 1.250 (0.645-2.421) | 0.491 (0.213-1.135) |
|  | Heavy drinkers | 6.089 (1.318-28.13) | 0.383 (0.125-1.171) |
| Demographic | |  |  |
|  | Age (Ref=60-69y) |  |  |
|  | 70+ | 3.840 (2.512-5.870) | 2.967 (1.817-4.845) |
|  | Gender (Ref=Female) |  |  |
|  | Male | 2.264 (1.249-4.105) | 2.941 (1.567-5.522) |
|  | Race (Ref= Non-Hispanic white) |  |  |
|  | Others (including multi-racial) | 0.825 (0.359-1.898) | 1.871 (0.834-4.199) |
|  | Other Hispanic | 3.936 (2.240-6.917) | 3.863 (1.095-13.62) |
|  | Mexican American | 2.398 (1.194-4.814) | 6.207 (3.145-12.24) |
|  | Non-Hispanic black | 5.055 (2.893-8.831) | 5.870 (2.829-12.17) |
|  | Education (Ref= College or above) |  |  |
|  | Under 12th grade | 6.064 (3.198-11.49) | 5.381 (2.776-10.43) |
|  | High school graduate | 1.897 (1.206-2.985) | 1.713 (0.860-3.411) |
|  | Marital status (Ref= Married/ Living with partner) |  |  |
|  | Widowed/ Divorced/ Separated | 2.448 (1.180-5.079) | 1.220 (0.657-2.266) |
|  | Never married | 1.399 (0.903-2.168) | 1.196 (0.510-2.802) |
|  | Income ratio (Ref= >3.50) |  |  |
|  | ≤1.30 | 2.448 (1.180-5.079) | 3.159 (1.673-5.966) |
|  | 1.31-3.50 | 1.399 (0.903-2.168) | 2.198 (1.166-4.143) |
| Lifestyle | |  |  |
|  | Smoking (Ref=Non-smokers) |  |  |
|  | Smokers | 0.940 (0.620-1.426) | 0.989 (0.637-1.535) |
|  | Physical activity (Ref= Active) |  |  |
|  | Inactive | 8.123 (1.959-33.68) | 0.621 (0.199-1.933) |
|  | BMI |  |  |
|  | Overweight | 0.519 (0.263-1.024) |  |
|  | Obesity | 0.563 (0.301-1.050) |  |
|  | Attend social events (Ref= No difficulty) |  |  |
|  | Difficulty | 2.226 (1.256-3.948) | 2.066 (1.030-4.142) |
| Comorbidities (Ref=Without) | |  |  |
|  | Stroke | 1.357 (0.639-2.881) | 1.175 (0.622-2.217) |
|  | CKD ^2^ | 2.264 (1.419-3.611) | 3.551 (1.828-6.897) |
| Interaction term | | p value | p value |
|  | Alcohol consumption* Physical activity | 0.007 | 0.037 |

^1^ Ref: reference. ^2^ CKD: Chronic Kidney Disease. ^3^ aOR: adjusted odds ratio. ^4^ CI: Confidence interval

Table S6. Multivariate logistic regression analyses of risk variables for association with cognitive impairment-stratified by Chronic kidney disease.

| **Variables** | | **Cognitive Impairment** | |
| --- | --- | --- | --- |
|  |  | **With CKD^2^** | **Without CKD** |
|  |  | **aOR^3^ (95% CI^4^)** | **aOR (95% CI)** |
| Alcohol consumption (Ref ^1^= Light drinkers) | |  |  |
|  | Moderate drinkers | 1.027 (0.506-2.085) | 0.867 (0.563-1.334) |
|  | Heavy drinkers | 6.324 (1.158-34.52) | 1.429 (0.486-4.204) |
| Demographic | |  |  |
|  | Age (Ref=60-69y) |  |  |
|  | 70+ | 4.526 (2.135-9.597) | 3.313 (2.378-4.617) |
|  | Gender (Ref=Female) |  |  |
|  | Male |  | 2.669 (1.727-4.124) |
|  | Race (Ref= Non-Hispanic white) |  |  |
|  | Others (including multi-racial) | 2.051 (0.494-8.508) | 1.167 (0.643-2.120) |
|  | Other Hispanic | 3.689 (0.904-15.05) | 3.879 (1.987-7.573) |
|  | Mexican American | 2.510 (0.839-7.510) | 3.439 (1.921-6.157) |
|  | Non-Hispanic black | 3.792 (1.778-8.086) | 5.181 (3.391-7.918) |
|  | Education (Ref= College or above) |  |  |
|  | Under 12th grade | 14.07 (6.583-30.09) | 4.546 (2.790-7.407) |
|  | High school graduate | 3.338 (1.744-6.390) | 1.428 (0.920-2.218) |
|  | Marital status (Ref= Married/ Living with partner) |  |  |
|  | Widowed/ Divorced/ Separated |  | 1.304 (0.832-2.043) |
|  | Never married |  | 0.567 (0.333-0.967) |
|  | Income ratio (Ref= >3.50) |  |  |
|  | ≤1.30 | 1.266 (0.475-3.377) | 2.765 (1.540-4.964) |
|  | 1.31-3.50 | 0.792 (0.380-1.649) | 1.854 (1.198-2.868) |
| Lifestyle | |  |  |
|  | Smoking (Ref=Non-smokers) |  |  |
|  | Smokers |  | 0.977 (0.735-1.300) |
|  | Physical activity (Ref= Active) |  |  |
|  | Inactive | 1.184 (0.573-2.444) | 1.520 (1.014-2.279) |
|  | Attend social events (Ref= No difficulty) |  |  |
|  | Difficulty | 4.451 (1.464-13.53) | 2.167 (1.049-4.479) |
| Comorbidities (Ref=Without) | |  |  |
|  | Hypertension |  | 1.611 (1.132-2.294) |
|  | Stroke | 3.136 (0.691-14.22) | 0.799 (0.329-1.942) |

^1^ Ref: reference. ^2^ CKD: Chronic Kidney Disease. ^3^ aOR: adjusted odds ratio. ^4^CI: Confidence interval

**Table S7.** Characteristics of participants (unweighted sample sizes and weighted %) according to alcohol consumption (drinks/day).

| **Alcohol Consumption, (drinks/day)** |  | **Total** | **1** | **2** | **3 to 4** | **5 to 6** | **7 to 8** | **9 to 10** | **>10** |
| --- | --- | --- | --- | --- | --- | --- | --- | --- | --- |
| ***Diabetes*** | | | | | | | | | |
| No of subjects |  | 573 | 335 | 126 | 70 | 25 | 7 | 4 | 6 |
| Alcohol (drinks/day), mean±SE |  | 1.97±1.847 | 1.00±0.000 | 2.00±0.000 | 3.36±0.483 | 5.44±0.507 | 7.57±0.535 | 10.00±0.000 | 12.67±1.633 |
| Cognitive function |  |  |  |  |  |  |  |  |  |
| Impairment, n (%) |  | 196 (34.2) | 97 (29.0) | 41 (32.5) | 36 (51.4) | 13 (52.0) | 5 (71.4) | 1 (25.0) | 3 (50.0) |
| DSST ^1^ score, mean±SE |  | 43.76±16.84 | 45.99±16.11 | 43.04±16.21 | 39.19±18.08 | 35.28±18.51 | 25.00±12.80 | 54.75±24.04 | 37.50±14.50 |
|  |  |  |  |  |  |  |  |  |  |
| ***Hypertension*** | | | | | | | | | |
| No of subjects |  | 1637 | 914 | 420 | 216 | 63 | 11 | 5 | 8 |
| Alcohol (drinks /day), mean±SE |  | 1.88±1.688 | 1.00±0.000 | 2.00±0.000 | 3.29±0.456 | 5.51±0.504 | 7.64±0.505 | 9.80±0.447 | 16.00±7.091 |
| Cognitive function |  |  |  |  |  |  |  |  |  |
| Impairment, n (%) |  | 492 (30.1) | 243 (26.6) | 113 (26.9) | 90 (41.7) | 35 (55.6) | 6 (54.5) | 1 (20.0) | 4 (50.0) |
| DSST ^1^ score, mean±SE |  | 46.88±17.23 | 47.95±16.72 | 48.18±17.49 | 43.33±18.03 | 38.48±16.55 | 35.64±9.521 | 47.20±20.42 | 34.63±13.876 |
|  |  |  |  |  |  |  |  |  |  |
| ***Chronic Kidney Disease (CKD)*** | | | | | | | | | |
| No of subjects |  | 520 | 279 | 124 | 79 | 30 | 3 | 2 | 3 |
| Alcohol (drinks/day), mean±SE |  | 2.00±1.661 | 1.00±0.000 | 2.00±0.000 | 3.34±0.477 | 5.43±0.504 | 8.00±0.000 | 10.00±0.000 | 13.33±2.309 |
| Cognitive function |  |  |  |  |  |  |  |  |  |
| Impairment, n (%) |  | 226 (43.5) | 109 (39.1) | 49 (39.5) | 40 (50.6) | 21 (70.0) | 3 (100.0) | 1 (50.0) | 3 (100.0) |
| DSST ^1^ score, mean±SE |  | 41.19±16.98 | 42.75±17.32 | 41.84±16.83 | 38.61±16.84 | 33.60±12.95 | 31.67±10.11 | 33.00±12.72 | 27.67±8.386 |

^1^ DSST: Digit Symbol Substitution Test.

**Table S8.** Odd ratio for cognitive impairment by different alcohol consumption categories-stratified by CKD.

| **Alcohol consumption** | **With CKD** | | | | | **Without CKD** | | | |
| --- | --- | --- | --- | --- | --- | --- | --- | --- | --- |
|  | **Model 1** | **Model 2** | **Model 3** | **Model 4^a^** | **Model 1** | | **Model 2** | **Model 3** | **Model 4^b^** |
|  | **OR (95%CI)** | **aOR (95%CI)** | **aOR (95%CI)** | **aOR (95%CI)** | **OR (95%CI)** | | **aOR (95%CI)** | **aOR (95%CI)** | **aOR (95%CI)** |
| 1 drink | Reference | Reference | Reference | Reference | Reference | | Reference | Reference | Reference |
| 2 drinks | 0.774  (0.424-1.411) | 0.522  (0.258-1.055) | 0.952  (0.503-1.803) | 0.618  (0.312-1.221) | 0.882  (0.622-1.251) | | 0.860  (0.568-1.304) | 0.908  (0.644-1.281) | 0.771  (0.488-1.217) |
| 3-4 drinks | 1.275  (0.632-2.572) | 1.206  (0.454-3.206) | 1.603  (0.804-3.196) | 1.354  (0.607-3.020) | 1.505  (1.018-2.226) | | 1.145  (0.640-2.047) | 1.564  (1.065-2.297) | 1.124  (0.588-2.149) |
| 5-6 drinks | 4.922  (1.735-13.96) | 4.485  (0.785-25.62) | 6.863  (2.168-21.55) | 4.988  (1.141-21.79) | 2.671  (1.322-5.395) | | 2.068  (0.720-5.937) | 2.634  (1.383-5.018) | 2.288  (0.660-7.929) |
| 7-8 drinks | 7.651^c^  (1.172-49.96) | 1.977  (0.142-27.44) | 8.215  (1.361-49.59) | 3.266  (0.258-41.29) | 4.623  (1.493-14.31) | | 0.539  (0.173-1.682) | 4.576  (1.410-14.85) | 1.250  (0.218-7.165) |
| 9-10 drinks |  |  |  |  | 0.332  (0.039-2.816) | | 0.337  (0.057-1.977) | 0.366  (0.042-3.190) | 0.168  (0.015-1.901) |
| 10+ drinks |  |  |  |  | 3.232  (0.741-14.10) | | 0.682  (0.079-5.919) | 3.611  (0.752-17.33) | 1.040  (0.131-8.280) |

CKD: Chronic Kidney Disease.

Model 1: crude OR (95%CI).

Model 2: adjusted for demographic variables.

Model 3: adjusted for comorbidity variables.

Model 4a: adjusted for all significant variables in the Table S6 (age, race, education).

Model 4b: adjusted for all significant variables in the Table S6 (age, gender, race, education, marriage, income, physical activities, social attendance, hypertension).

^C^:Due to the small number of subject, more than or equal to 7 drinks are combined and presented together.

# Supplementary Figure


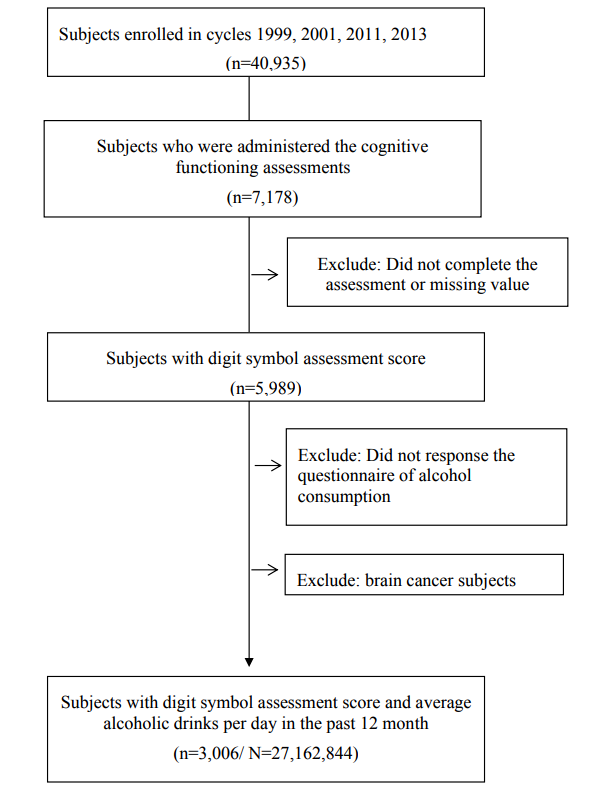


**Supplementary Figure 1.** Flow chart of subject selection.
